# Associations between chemoradiotherapy-based immune checkpoint blockade and posttreatment depression and anxiety in head and neck cancer patients: a cross-sectional study

**DOI:** 10.3389/fimmu.2025.1649486

**Published:** 2025-09-01

**Authors:** Yang Zhang, Yunhao Chen, Xianxing Zhang, Yafang Hong, Zihan Zhou, Xingchen Ding

**Affiliations:** ^1^ Clinical Diagnosis and Treatment Center of Oncology, The Second Affiliated Hospital of Soochow University, Suzhou, China; ^2^ Institute of Radiotherapy & Oncology, Soochow University, Suzhou, China; ^3^ Department of Radiation Oncology, The Affiliated Cancer Hospital of Nanjing Medical University and Jiangsu Cancer Hospital and Jiangsu Institute of Cancer Research, Nanjing, China; ^4^ Department of Otolaryngology, Affiliated Hospital of Shandong University of Traditional Chinese Medicine, Jinan, China; ^5^ Department of Radiation Oncology, Fujian Medical University Union Hospital, Fuzhou, China; ^6^ Department of Basic Medical Sciences, 960th Hospital of People's Liberation Army Joint Logistic Support Force, Jinan, China; ^7^ Department of Radiation Oncology, Shandong Cancer Hospital and Institute, Shandong First Medical University and Shandong Academy of Medical Sciences, Jinan, Shandong, China

**Keywords:** immune checkpoint blockade, head and neck cancer, chemoradiotherapy, depression, anxiety

## Abstract

Immune checkpoint blockade (ICB) and combination treatment paradigms have gradually improved the prognosis of head and neck cancer (HNC) patients. However, HNC survivors struggle with anxiety and depression because of the large variety of persistent cancer-related or treatment-related symptoms. Recent studies have suggested that emotional distress is closely related to the therapeutic efficacy of ICB. In this study, 232 advanced HNC patients were recruited and their anxiety and depression status were assessed by the Hospital Anxiety and Depression Scale (HADS). Propensity score matching (PSM) was applied to balance confounders. The nearest-neighbor matching method was used for PSM matching to perform 1:1 matching. By comparing the anxiety and depression status of HNC patients after PSM, we showed that HNC patients receiving chemoradiotherapy (CRT) combined with ICB, both concurrent radiotherapy (RT) and sequential RT, had lower depression levels than did those receiving CRT alone. These findings suggest that ICB treatment may be associated with emotional status, which may could offer insights into ameliorate quality of life, both physically and psychologically, of HNC patients.

## Graphical Abstract

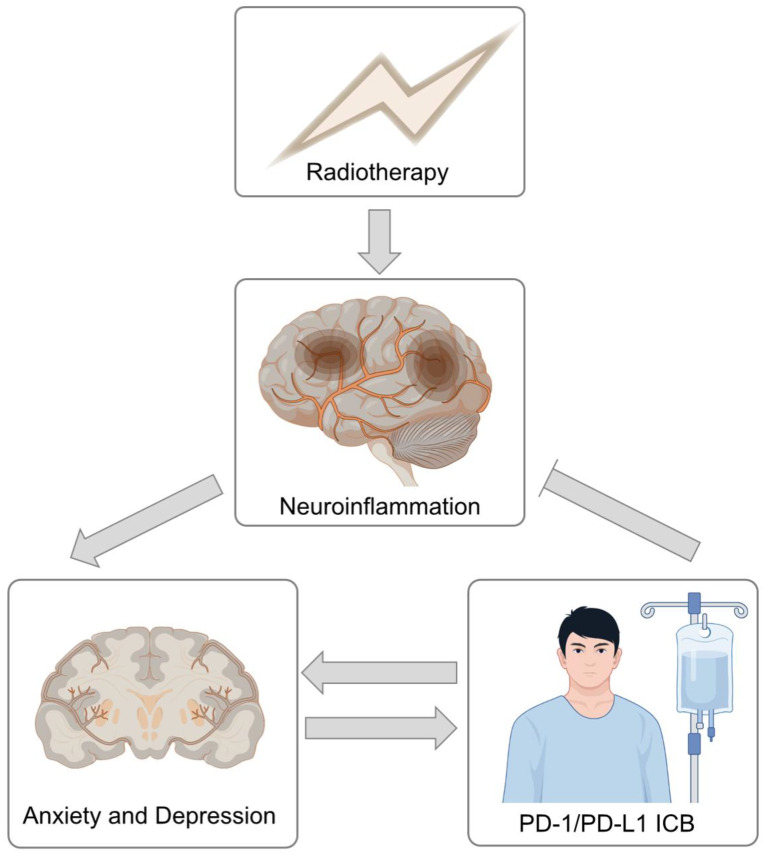

## Introduction

The incidence of head and neck cancer (HNC) has risen to sixth place among all tumors worldwide (1, 2). Radiotherapy (RT), which is a traditional antitumor method, plays an irreplaceable role in HNC treatment. The role of immune checkpoint blockade (ICB) in the treatment of HNC is increasing. The combination of RT and ICB has been hailed as a new dawn for antitumor treatment (3). The long-term effects of treatment are of concern in survivors. Most studies have shown that HNC survivors struggle with distress and depression in addition to a large variety of persistent cancer-related symptoms (4, 5). Higher rates of anxiety and depression symptoms are found in patients with HNC because of their unique tumor location (6). Integrated supportive care, especially cognitive-behavioral therapy (CBT) and mindfulness-based interventions, could help patients identify and modify negative thought patterns and behaviors and thus alleviating psychological distress (7, 8). Notably, anxiety and depression in these patients not only diminish quality of life but also might affect antitumor therapeutic efficacy. For example, Zeng et al. and Fraterman et al. reported an association between emotional distress and worse clinical outcomes in advanced non-small cell lung cancer and melanoma patients receiving ICB treatment (9, 10). This finding indicated an intrinsic link between emotional distress and ICB treatment. On the one hand, the undifferentiated attack of ICB often induces immune-related adverse events. On the other hand, immune checkpoint blockade might activate common immune-dependent repair mechanisms. However, to our knowledge, no studies have investigated the associations of the combination of RT and ICB with depression and anxiety in HNC patients.

## Methods

Between October 9, 2020, and December 6, 2023, stage III-IV HNC patients undergoing intensity-modulated radiotherapy (IMRT) were recruited from Shandong Cancer Hospital and Institute. All male and female participants provided informed consent. The inclusion criteria were as follows: (1) new diagnosis of stage III-IV stage head and neck cancer (nasopharynx, larynx, oropharynx, or hypopharynx) according to the eighth AJCC and pathological staging; and (2) concurrent chemotherapy and radiotherapy treatment. (3) Combined ICB patients must receive ICB therapy (PD-1 and PD-L1 inhibitors) during or before RT. (4) Control patients must never receive ICB therapy before or after RT. The exclusion criteria were as follows: (1) had multiple primary cancers, (2) had prior irradiation, and (3) could not cooperate with the relevant evaluation and follow-up of the study.

All patients received daily IMRT treatment using 2.0 Gy fractions. The total dose range was 48–76 Gy. Patients were divided into 3 groups: RT + chemotherapy ± targeted drug group (control group), ICB + sequential RT + chemotherapy ± targeted drug group (ICB + sRT group) and RT + chemotherapy ± targeted drug + concurrent ICB group (ICB + cRT group). ICB drugs include PD-1 inhibitors (camrelizumab, pembrolizumab, toripalimab, sintilimab, tislelizumab) and a PD-L1 inhibitor (adebrelimab). The main outcomes considered were anxiety and depression scores on the Hospital Anxiety and Depression Scale (HADS) (11). Originally designed as a self-assessment tool for detecting depression and anxiety, the HADS is now extensively utilized in both nonhospital settings and research studies. The assessment includes 14 questions, with seven evaluating anxiety and seven evaluating depression, resulting in two subscale scores. Each question is rated on a scale from 0 to 3, where a higher score signifies a greater severity of mood disorder. No missing data were present for HADS scores or covariates, and a complete-case approach was naturally applied. Patients completed questionnaires by email, postal mail or phone on Feb. 01, 2024. We adopted 1:1 propensity score matching (PSM) via the nearest-neighbor matching method to minimize between-group heterogeneity and selection bias for the control group vs. the ICB + sRT group, the control group vs. the ICB + cRT group, and the ICB + sRT group vs. the ICB + cRT group. The caliper width was set at 0.2 standard deviations, with no replacement applied. The study’s propensity score incorporated the following variables: time since radiotherapy, DT, tumor type, gender, age, KPS score, smoking status, alcohol consumption, BMI, coronary artery disease (CAD), hypertension, diabetes, T stage, N stage, M stage, and target drug. Pearson’s chi-square test (or Fisher’s exact test for sparse data) was used to compare categorical variables between the two groups. Multiple comparisons were corrected using the Bonferroni-Holm correction. The effect size (such as r value), power (80%), and significance level (α = 0.05) used to determine the required sample size. Independent samples t tests or Wilcoxon rank-sum tests (depending on distributional assumptions) were used to compare the differences in anxiety and depression scores between two groups. p-values were adjusted using the Holm-Bonferroni correction (Holm, 1979). The steps for the power analysis are as follows: convert the effect size r value to the corresponding Z-value, calculate the noncentrality parameter by incorporating the sample size and significance level, and then back-calculate the power. Quantitative data with a normal distribution are shown as the means ± SDs; if not normally distributed, they are represented as medians (Q1, Q3). The date were analyzed using SPSS (v26.0) and R software (v4.2.3). Graphs were created using GraphPad Prism (v10.1.2). P <0.05 was considered statistically significant.

## Results

### Demographics

**Figure 1:**
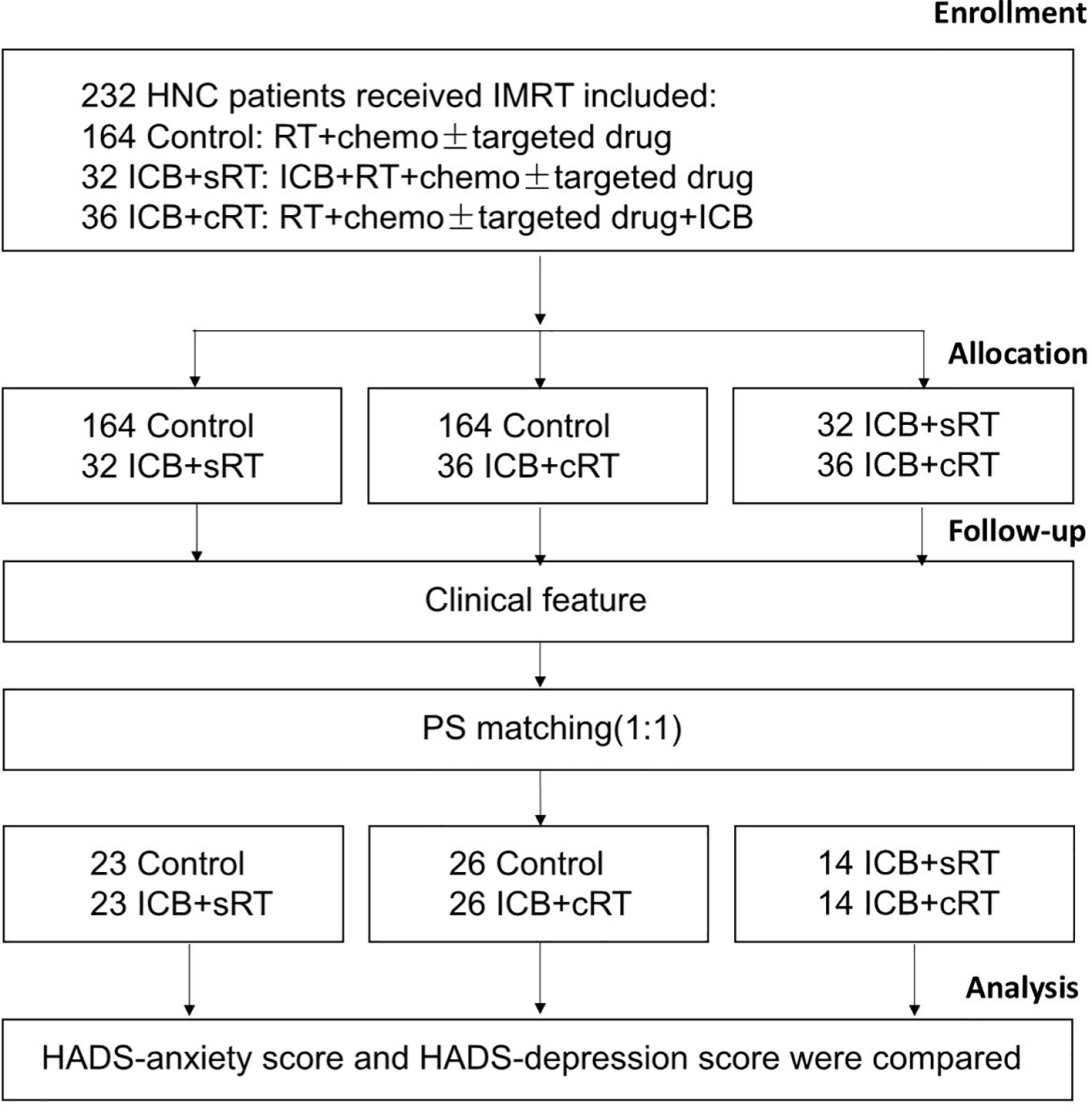
The research flow chart.

The research flowchart and PSM protocol are shown in **Figure 1**. After PSM, no significant difference remained in the baseline characteristics of the matched patients between the two groups (**Tables 1**–**3**). Twenty-three patients in the control group were matched with 23 patients in the ICB + sRT group (**Table 1**). Twenty-six patients in the control group were matched with 26 patients in the ICB + cRT group (**Table 2**). Fourteen patients in the ICB + sRT group were matched with 14 patients in the ICB + cRT group (**Table 3**). Love plots to demonstrate covariate balance were shown in the **Supplementary Figure 1**.

**Table 1:** Patients characters before and after PSM (Control group vs. ICB + sRT group).

Variables	Before PSM				After PSM			
	Control, n=164	ICB+sRT, n=32	P value	SMD	Control, n=23	ICB+sRT, n=23	P value	SMD
Tumor type, n (%)			0.001	0.701			0.605	0.208
Nasopharyngeal carcinoma	79 (48.171)	6 (18.750)			4 (17.391)	5 (21.739)		
Laryngocarcinoma	34 (20.732)	7 (21.875)			5 (21.739)	5 (21.739)		
Oropharynx carcinoma	12 (7.317)	4 (12.500)			2 (8.696)	3 (13.043)		
Hypopharyngeal carcinoma	39 (23.780)	15 (46.875)			12 (52.174)	10 (43.478)		
Gender, n (%)			0.135	0.413			1.000	0.177
Male	140 (85.366)	31 (96.875)			21 (91.304)	22 (95.652)		
Female	24 (14.634)	1 (3.125)			2 (8.696)	1 (4.348)		
Age, n (%)			0.010	0.575			0.601	0.312
<50	52 (31.707)	3 (9.375)			1 (4.348)	3 (13.043)		
≥50	112 (68.293)	29 (90.625)			22 (95.652)	20 (86.957)		
KPS Score, n (%)			0.372	0.180			1.000	0.110
<90	43 (26.220)	6 (18.750)			4 (17.391)	5 (21.739)		
≥90	121 (73.780)	26 (81.250)			19 (82.609)	18 (78.261)		
Smoking, n (%)			0.014	0.491			0.753	0.093
Yes	90 (54.878)	10 (31.250)			7 (30.435)	8 (34.783)		
No	74 (45.122)	22 (68.750)			16 (69.565)	15 (65.217)		
Alcohol, n (%)			0.007	0.539			0.345	0.281
Yes	99 (60.366)	11 (34.375)			6 (26.087)	9 (39.130)		
No	65 (39.634)	21 (65.625)			17 (73.913)	14 (60.870)		
BMI, n (%)			0.390	0.206			0.697	0.231
<25	141 (85.976)	25 (78.125)			18 (78.261)	20 (86.957)		
≥25	23 (14.024)	7 (21.875)			5 (21.739)	3 (13.043)		
CAD, n (%)			0.431	0.320			1.000	0.000
Yes	8 (4.878)	0 (0.000)			0 (0.000)	0 (0.000)		
No	156 (95.122)	32 (100.000)			23 (100.000)	23 (100.000)		
Hypertension, n (%)			0.144	0.267			1.000	0.000
Yes	28 (17.073)	9 (28.125)			6 (26.087)	6 (26.087)		
No	136 (82.927)	23 (71.875)			17 (73.913)	17 (73.913)		
Diabetes, n (%)			0.446	0.204			1.000	0.000
Yes	7 (4.268)	3 (9.375)			2 (8.696)	2 (8.696)		
No	157 (95.732)	29 (90.625)			21 (91.304)	21 (91.304)		
T stage, n (%)			0.796	0.426			0.863	0.109
T1	11 (6.707)	5 (15.625)			2 (8.696)	2 (8.696)		
T2	49 (29.878)	7 (21.875)			6 (26.087)	7 (30.435)		
T3	70 (42.683)	10 (31.250)			9 (39.130)	8 (34.783)		
T4	34 (20.732)	10 (31.250)			6 (26.087)	6 (26.087)		
N stage, n (%)			0.953	0.265			0.612	0.262
N0	21 (12.805)	6 (18.750)			7 (30.435)	5 (21.739)		
N1	24 (14.634)	4 (12.500)			1 (4.348)	2 (8.696)		
N2	89 (54.268)	14 (43.750)			11 (47.826)	11 (47.826)		
N3	30 (18.293)	8 (25.000)			4 (17.391)	5 (21.739)		
M stage, n (%)			0.002	0.504			1.000	0.140
M0	161 (98.171)	27 (84.375)			21 (91.304)	20 (86.957)		
M1	3 (1.829)	5 (15.625)			2 (8.696)	3 (13.043)		
Target drug, n (%)			0.555	0.211			1.000	0.000
Yes	13 (7.927)	1 (3.125)			1 (4.348)	1 (4.348)		
No	151 (92.073)	31 (96.875)			22 (95.652)	22 (95.652)		
Time since radiotherapy (day), median (Q1, Q3)	540.500 (198.250, 916.500)	244.500 (109.500, 594.250)	0.001	0.664	290.000 (167.500, 474.000)	274.000 (130.500, 637.000)	0.728	0.016
DT (Gy), Mean ± SD	68.133 ± 4.313	64.625 ± 6.880	0.009	0.619	67.043 ± 5.966	67.478 ± 4.187	0.776	0.086

**Table 2:** Patients characters before and after PSM (Control group vs. ICB + cRT group).

Variables	Before PSM				After PSM			
	Control, n=164	ICB+cRT, n=36	P value	SMD	Control, n=26	ICB+cRT, n=26	P value	SMD
Tumor type, n (%)			0.003	0.672			0.763	0.265
Nasopharyngeal carcinoma	79 (48.171)	11 (30.556)			4 (15.385)	6 (23.077)		
Laryngocarcinoma	34 (20.732)	3 (8.333)			3 (11.538)	3 (11.538)		
Oropharynx carcinoma	12 (7.317)	3 (8.333)			5 (19.231)	3 (11.538)		
Hypopharyngeal carcinoma	39 (23.780)	19 (52.778)			14 (53.846)	14 (53.846)		
Gender, n (%)			0.233	0.305			1.000	0.166
Male	140 (85.366)	34 (94.444)			25 (96.154)	24 (92.308)		
Female	24 (14.634)	2 (5.556)			1 (3.846)	2 (7.692)		
Age, n (%)			0.261	0.215			0.482	0.196
<50	52 (31.707)	8 (22.222)			4 (15.385)	6 (23.077)		
≥50	112 (68.293)	28 (77.778)			22 (84.615)	20 (76.923)		
KPS Score, n (%)			0.596	0.096			0.510	0.183
<90	43 (26.220)	11 (30.556)			5 (19.231)	7 (26.923)		
≥90	121 (73.780)	25 (69.444)			21 (80.769)	19 (73.077)		
Smoking, n (%)			0.082	0.325			0.388	0.241
Yes	90 (54.878)	14 (38.889)			8 (30.769)	11 (42.308)		
No	74 (45.122)	22 (61.111)			18 (69.231)	15 (57.692)		
Alcohol, n (%)			0.019	0.440			0.388	0.241
Yes	99 (60.366)	14 (38.889)			8 (30.769)	11 (42.308)		
No	65 (39.634)	22 (61.111)			18 (69.231)	15 (57.692)		
BMI, n (%)			0.410	0.146			1.000	0.113
<25	141 (85.976)	29 (80.556)			23 (88.462)	22 (84.615)		
≥25	23 (14.024)	7 (19.444)			3 (11.538)	4 (15.385)		
CAD, n (%)			1.000	0.030			1.000	0.131
Yes	8 (4.878)	2 (5.556)			3 (11.538)	2 (7.692)		
No	156 (95.122)	34 (94.444)			23 (88.462)	24 (92.308)		
Hypertension, n (%)			0.010	0.441			0.768	0.082
Yes	28 (17.073)	13 (36.111)			8 (30.769)	9 (34.615)		
No	136 (82.927)	23 (63.889)			18 (69.231)	17 (65.385)		
Diabetes, n (%)			0.220	0.259			1.000	0.131
Yes	7 (4.268)	4 (11.111)			3 (11.538)	2 (7.692)		
No	157 (95.732)	32 (88.889)			23 (88.462)	24 (92.308)		
T stage, n (%)			0.374	0.644			0.914	0.173
T1	11 (6.707)	3 (8.333)			1 (3.846)	2 (7.692)		
T2	49 (29.878)	12 (33.333)			10 (38.462)	9 (34.615)		
T3	70 (42.683)	6 (16.667)			4 (15.385)	4 (15.385)		
T4	34 (20.732)	15 (41.667)			11 (42.308)	11 (42.308)		
N stage, n (%)			0.010	0.518			0.830	0.321
N0	21 (12.805)	1 (2.778)			0 (0.000)	1 (3.846)		
N1	24 (14.634)	3 (8.333)			3 (11.538)	2 (7.692)		
N2	89 (54.268)	20 (55.556)			17 (65.385)	16 (61.538)		
N3	30 (18.293)	12 (33.333)			6 (23.077)	7 (26.923)		
M stage, n (%)			<0.001	0.847			1.000	0.113
M0	161 (98.171)	25 (69.444)			23 (88.462)	22 (84.615)		
M1	3 (1.829)	11 (30.556)			3 (11.538)	4 (15.385)		
Target drug, n (%)			0.889	0.095			1.000	0.283
Yes	13 (7.927)	2 (5.556)			0 (0.000)	1 (3.846)		
No	151 (92.073)	34 (94.444)			26 (100.000)	25 (96.154)		
Time since radiotherapy (day), median (Q1, Q3)	540.500 (198.250, 916.500)	236.000 (160.000, 552.500)	0.053	0.304	290.000 (194.000, 505.250)	271.000 (180.500, 737.750)	0.749	0.293
DT (Gy), Mean ± SD	68.133 ± 4.313	66.900 ± 5.672	0.226	0.247	68.077 ± 3.939	66.938 ± 5.921	0.418	0.231

**Table 3:** Patients characters before and after PSM (ICB + sRT group vs. ICB + cRT group).

Variables	Before PSM				After PSM			
	ICB+sRT, n=32	ICB+cRT, n=36	P value	SMD	ICB+sRT, n=14	ICB+cRT, n=14	P value	SMD
Tumor type, n (%)			0.958	0.461			0.707	0.155
Nasopharyngeal carcinoma	6 (18.750)	11 (30.556)			6 (42.857)	5 (35.714)		
Laryngocarcinoma	7 (21.875)	3 (8.333)			1 (7.143)	1 (7.143)		
Oropharynx carcinoma	4 (12.500)	3 (8.333)			1 (7.143)	1 (7.143)		
Hypopharyngeal carcinoma	15 (46.875)	19 (52.778)			6 (42.857)	7 (50.000)		
Gender, n (%)			1.000	0.119			1.000	0.000
Male	31 (96.875)	34 (94.444)			13 (92.857)	13 (92.857)		
Female	1 (3.125)	2 (5.556)			1 (7.143)	1 (7.143)		
Age, n (%)			0.151	0.358			0.596	0.417
<50	3 (9.375)	8 (22.222)			3 (21.429)	1 (7.143)		
≥50	29 (90.625)	28 (77.778)			11 (78.571)	13 (92.857)		
KPS Score, n (%)			0.262	0.277			1.000	0.166
<90	6 (18.750)	11 (30.556)			3 (21.429)	4 (28.571)		
≥90	26 (81.250)	25 (69.444)			11 (78.571)	10 (71.429)		
Smoking, n (%)			0.511	0.161			1.000	0.166
Yes	10 (31.250)	14 (38.889)			4 (28.571)	3 (21.429)		
No	22 (68.750)	22 (61.111)			10 (71.429)	11 (78.571)		
Alcohol, n (%)			0.700	0.094			1.000	0.000
Yes	11 (34.375)	14 (38.889)			4 (28.571)	4 (28.571)		
No	21 (65.625)	22 (61.111)			10 (71.429)	10 (71.429)		
BMI, n (%)			0.805	0.060			1.000	0.187
<25	25 (78.125)	29 (80.556)			11 (78.571)	12 (85.714)		
≥25	7 (21.875)	7 (19.444)			3 (21.429)	2 (14.286)		
CAD			0.494	0.343			1.000	0.000
Yes	0 (0.000)	2 (5.556)			0 (0.000)	0 (0.000)		
No	32 (100.000)	34 (94.444)			14 (100.000)	14 (100.000)		
Hypertension, n (%)			0.482	0.172			1.000	0.166
Yes	9 (28.125)	13 (36.111)			4 (28.571)	3 (21.429)		
No	23 (71.875)	23 (63.889)			10 (71.429)	11 (78.571)		
Diabetes, n (%)			1.000	0.057			1.000	0.000
Yes	3 (9.375)	4 (11.111)			1 (7.143)	1 (7.143)		
No	29 (90.625)	32 (88.889)			13 (92.857)	13 (92.857)		
T stage, n (%)			0.603	0.471			0.981	0.764
T1	5 (15.625)	3 (8.333)			2 (14.286)	2 (14.286)		
T2	7 (21.875)	12 (33.333)			3 (21.429)	5 (35.714)		
T3	10 (31.250)	6 (16.667)			5 (35.714)	1 (7.143)		
T4	10 (31.250)	15 (41.667)			4 (28.571)	6 (42.857)		
N stage, n (%)			0.090	0.575			0.740	0.478
N0	6 (18.750)	1 (2.778)			0 (0.000)	1 (7.143)		
N1	4 (12.500)	3 (8.333)			2 (14.286)	1 (7.143)		
N2	14 (43.750)	20 (55.556)			7 (50.000)	8 (57.143)		
N3	8 (25.000)	12 (33.333)			5 (35.714)	4 (28.571)		
M stage, n (%)			0.147	0.360			1.000	0.166
M0	27 (84.375)	25 (69.444)			10 (71.429)	11 (78.571)		
M1	5 (15.625)	11 (30.556)			4 (28.571)	3 (21.429)		
Target drug, n (%)			1.000	0.119			1.000	0.000
Yes	1 (3.125)	2 (5.556)			1 (7.143)	1 (7.143)		
No	31 (96.875)	34 (94.444)			13 (92.857)	13 (92.857)		
Time since radiotherapy (day), median (Q1, Q3)	244.500 (109.500, 594.250)	236.000 (160.000, 552.500)	0.332	0.278	576.000 (193.000, 701.500)	196.500 (156.250, 441.250)	0.154	0.412
DT (Gy), Mean ± SD	64.625 ± 6.880	66.900 ± 5.672	0.140	0.366	66.571 ± 4.536	67.571 ± 4.972	0.583	0.218

### HADS score

**Figure 2:**
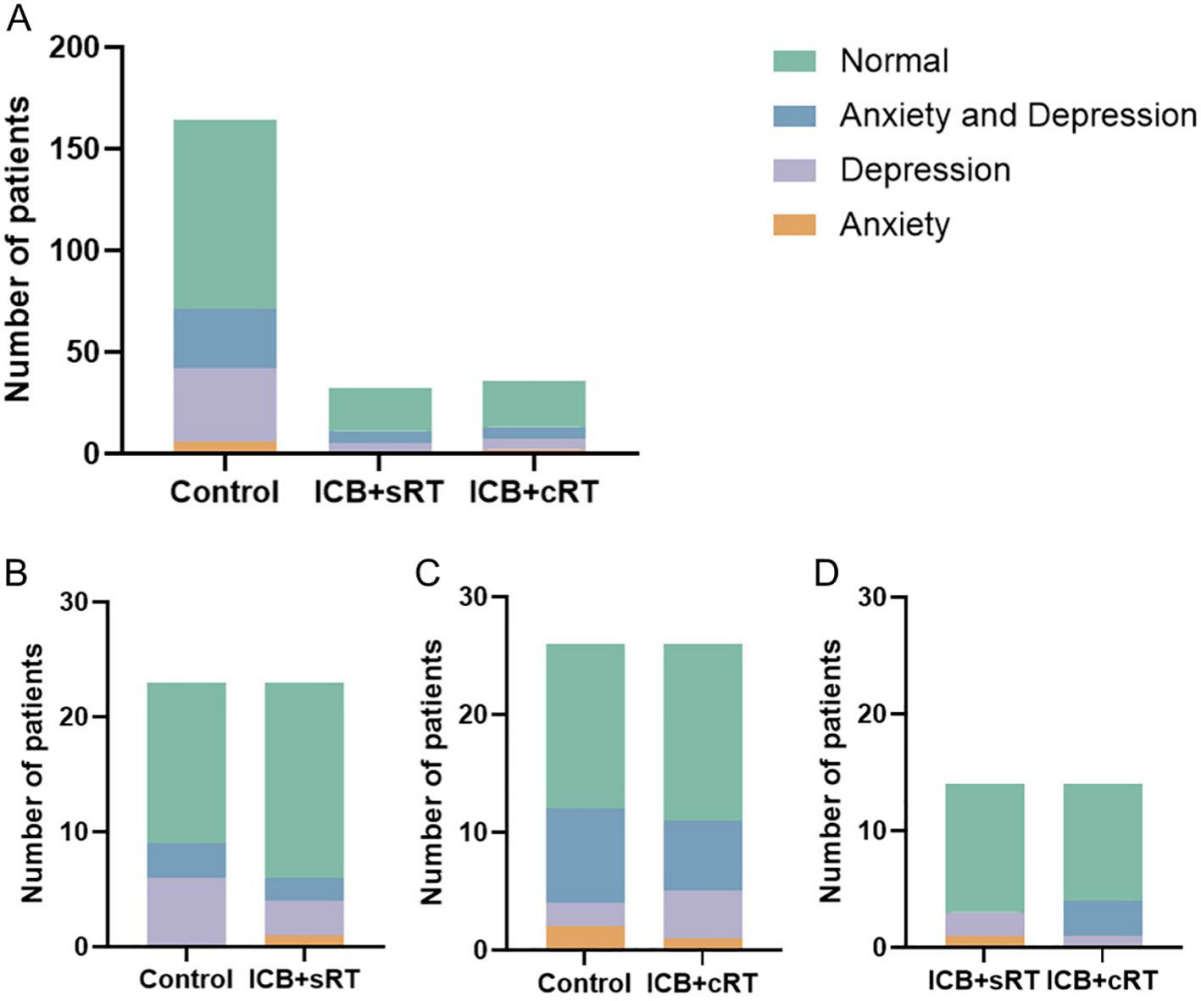
The distribution of patients with anxiety and depression. **(A)** The distribution of patients with anxiety and depression in the control group (n = 164), the ICB + sRT group (n = 32), and the ICB + cRT group (n = 36) before PSM. **(B–D)** The distribution of patients with anxiety and depression between the control group and the ICB + sRT group (n = 23), the control group and the ICB + cRT group (n = 26), and the ICB + sRT group and the ICB + cRT group (n = 14) after PSM.

A total of 232 patients were included, and the numbers of patients with anxiety in the control group, ICB + sRT group, and ICB + cRT group were 6, 1, and 2, respectively; the numbers of patients with depression were 36, 4, and 5, respectively; the numbers of patients with both anxiety and depression were 29, 6, and 6, respectively; and the occurrence rates of anxiety or depression were 43.29% (71/164), 34.38% (11/32), and 36.11% (13/36), respectively (**Figure 2A**). After PSM, the occurrence rates of anxiety or depression were 39.13% (9/23) and 26.09% (6/23) in the control group and the ICB + sRT group, respectively (**Figure 2B**). The occurrence rates of anxiety or depression were 46.15% (12/26) and 42.31% (11/26) in the control group and the ICB + cRT group, respectively (**Figure 2C**). The occurrence rates of anxiety or depression were 21.43% (3/14) and 28.57% (4/14) in the ICB + sRT group and the ICB + cRT group, respectively (**Figure 2D**).

### Association of ICB with HADS score

**Figure 3:**
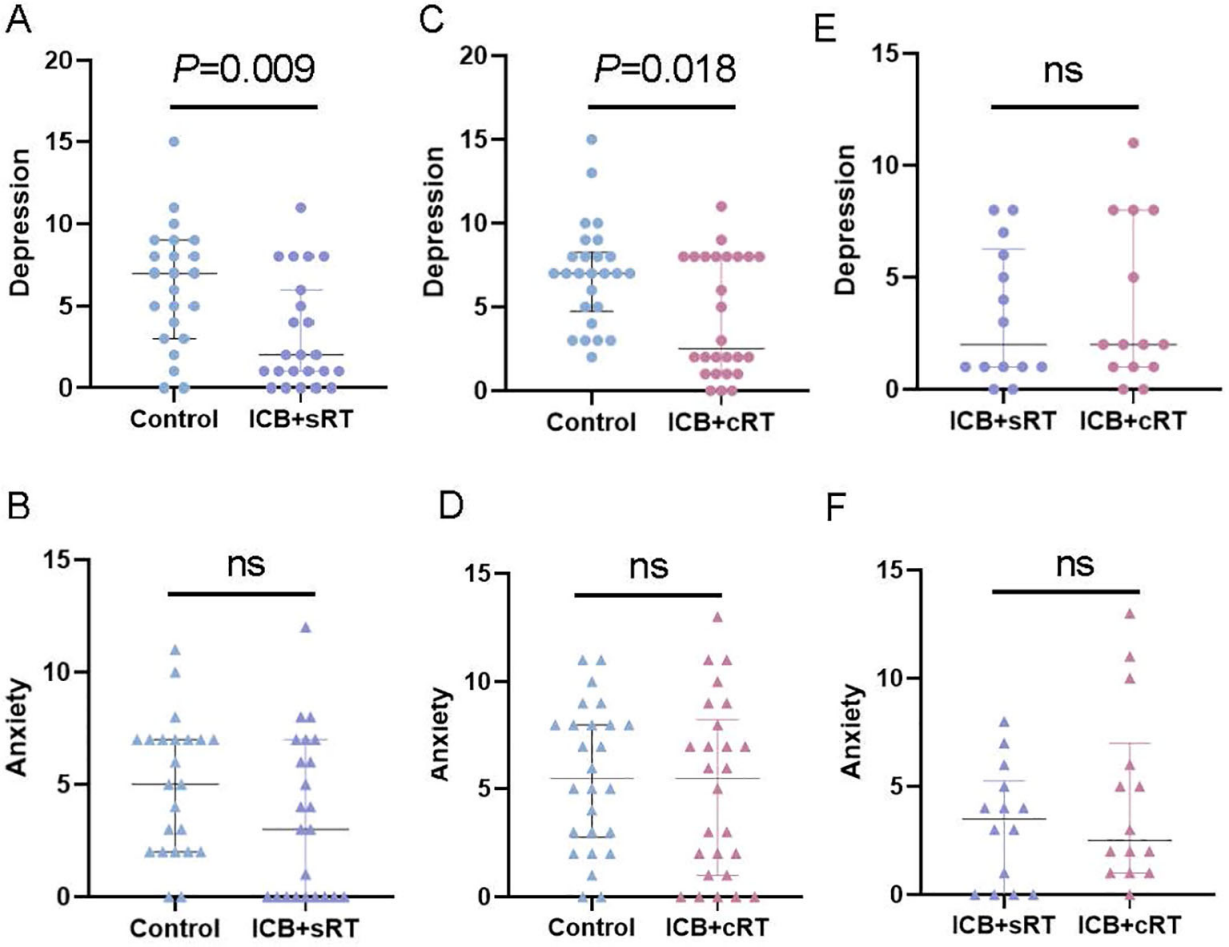
Association of ICB with HADS scores. **(A–E)** Comparison of depression status between the control group and the ICB + sRT group, the control group and the ICB + cRT group, and the ICB + sRT group and the ICB + cRT group. **(B–F)** Comparison of anxiety status between the control group and the ICB + sRT group, the control group and the ICB + cRT group, and the ICB + sRT group and the ICB + cRT group. The median and interquartile range of anxiety and depression scores are shown by bars. ns, not significant.

After PSM, compared with those of the control group, the depression scores of the ICB + sRT group were significantly lower than those of the control group [2.00 (1.00, 6.00) vs. 7.00 (3.00, 9.00), P = 0.009; r = 0.386, 95% CI (0.121-0.624), power = 0.82; **Figure 3A**]. However, the anxiety scores of the ICB + sRT group did not differ from those of the control group [3.00 (0.00, 7.00) vs. 5.00 (2.00, 7.00), P = 0.124; r = 0.227, 95% CI (-0.053-0.499), power = 0.35; **Figure 3B**]. Similarly, the depression scores of the combined ICB + cRT group were significantly lower [2.50 (1.00, 8.00) vs. 7.00 (4.75, 8.25), P = 0.018; r = 0.327, 95% CI (0.041-0.562), power = 0.68; **Figure 3C**]. However, no difference was detected in the anxiety scores between the two groups [5.50 (1.00, 8.25) vs. 5.50 (2.75, 8.00), P = 0.473; r = 0.099, 95% CI (-0.179-0.374), power = 0.15; **Figure 3D**]. For the ICB + sRT group and the ICB + cRT group, no differences in depression scores [2.00 (1.00, 6.25) vs. 2.00 (1.00, 8.00), P = 0.708; r = 0.071, 95% CI (-0.312-0.460), power = 0.09; **Figure 3E**] or anxiety scores [3.50 (0.00, 5.25) vs. 2.50 (1.00, 7.00), P = 0.548; r = 0.114, 95% CI (-0.267-0.488); power = 0.12, **Figure 3F**] were detected.

## Discussion

This is the first study to explore the effect of combined ICB on depression and anxiety in stage III–IV HNC patients after RT. We found that HNC patients receiving CRT combined with ICB, both concurrent RT and sequential RT, had lower depression levels than did patients receiving CRT alone. As previously mentioned, two studies have reported the negative roles of anxiety and depression in the efficacy of ICB. It looks like a closed loop (graphical abstract) (9, 10). There is currently no direct evidence to explain this result. It has previously been proposed that increased depression is independently linked to worsened neurocognition among primary brain tumor patients receiving RT (12). For late-stage HNC patients, the target area of RT is relatively large and normal brain tissue is inevitably inevitable exposed to radiation. Clinical manifestations may include mental disorders, focal neurological deficits, and the progressive degeneration of learning and memory functions due to hippocampal damage (13–16). Therefore, one of the possible explanations might be related to the effect of PD-1/PD-L1 inhibitors on the cognitive function of patients. Alzheimer’s disease (AD) is clinically characterized by memory loss and cognitive decline (17). In AD and dementia mouse models, Baruch et al. and Rosenzweig et al. demonstrated that a PD-1/PD-L1 inhibitor evokes an IFNγ-dependent systemic immune response that recruits monocyte-derived macrophages to the brain. This immunological response can improve cognitive performance (18, 19). However, a more likely explanation is that, PD-1 inhibitors can reduce neuroinflammatory responses and may also regulate neuronal activity (19–21). Direct damage to neurons and chronic inflammation induced by RT are well recognized (22), and depressive-like behavior *in vivo* occurs mainly via neuroinflammatory response activation (23). It is reasonable to speculate that PD-1/PD-L1 inhibitors can also reduce the RT-induced decrease in cognitive function. However, owing to the lack of brain structure-specific dosimetry data, especially for hippocampal radiation exposure, we cannot directly assess the relationships among regional radiation doses, neuroinflammation, and changes in anxiety or depression. These findings should be further validated in HNC patients.

Additionally, although our findings revealed a statistically detectable association between radiotherapy sequencing data and HADS scores, the magnitude of these differences may not reach the threshold for meaningful clinical changes in most patients. The clinical implications of these unique findings merit further evaluation, and pretreatment mood assessment should be included to assess treatment-attributable changes in future studies. Notably, power analysis revealed that after PSM, the statistical power for some comparisons fell below 0.8, indicating that our ability to detect true differences in these cases was limited, with an increased risk of Type II error. Future studies with larger sample sizes would help strengthen the evidentiary basis and confirm the robustness of these findings.

### Limitations

This study had several limitations. First, our study is subject to potential survivorship bias and immortal-time bias, which may have excluded those with worse clinical or psychological outcomes, potentially skewing our findings toward more favorable mood profiles. Second, this was a single-center study without independent validation. Third, the types of anti-PD-1/PD-L1 antibodies used for treatment were heterogeneous, subgroup analyses were not feasible here given the individualized nature of drug selection and small sample sizes for certain agents, which would compromise statistical robustness. Furthermore, the omission of key confounders, including socioeconomic status, education, social support, psychotropic medication use, and prior psychiatric history, may introduce residual confounding, as they could independently influence anxiety or depression scores and thus affect the interpretation of our findings. Multicenter and prospective studies are needed to confirm these findings.

## Conclusion

In this study, HNC patients receiving CRT combined with ICB, both concurrent RT and sequential RT, had lower depression levels than did those receiving CRT alone. Conversely, no significant differences in anxiety levels were noted across these treatment groups. This study is the first to show that patients who received ICB tended to have lower HADS scores over time. This information may have important implications for personalized approaches to preventing anxiety or depression in HNC patients.

## Data Availability

The raw data supporting the conclusions of this article will be made available by the authors, without undue reservation.

## Supplementary material

Supplementary Figure 1 The Love plots before and after PSM. **(A, B)**: Love plots of the control group and the ICB + sRT group before and after PSM. **(C, D)**: Love plots of the control group and the ICB + cRT group before and after PSM. **(E, F)**: Love plots of the ICB + sRT group and the ICB + cRT group before and after PSM. Group 1: The control group. Group 2: The ICB + sRT group. Group 3: the ICB + cRT group.
