# Supplementary figures and images for: Associations between chemoradiotherapy-based immune checkpoint blockade and posttreatment depression and anxiety in head and neck cancer patients: a cross-sectional study
Source: Front Immunol. 2025 Sep 1;16:1649486. doi: 10.3389/fimmu.2025.1649486 (PMC12434114; doi:10.3389/fimmu.2025.1649486)

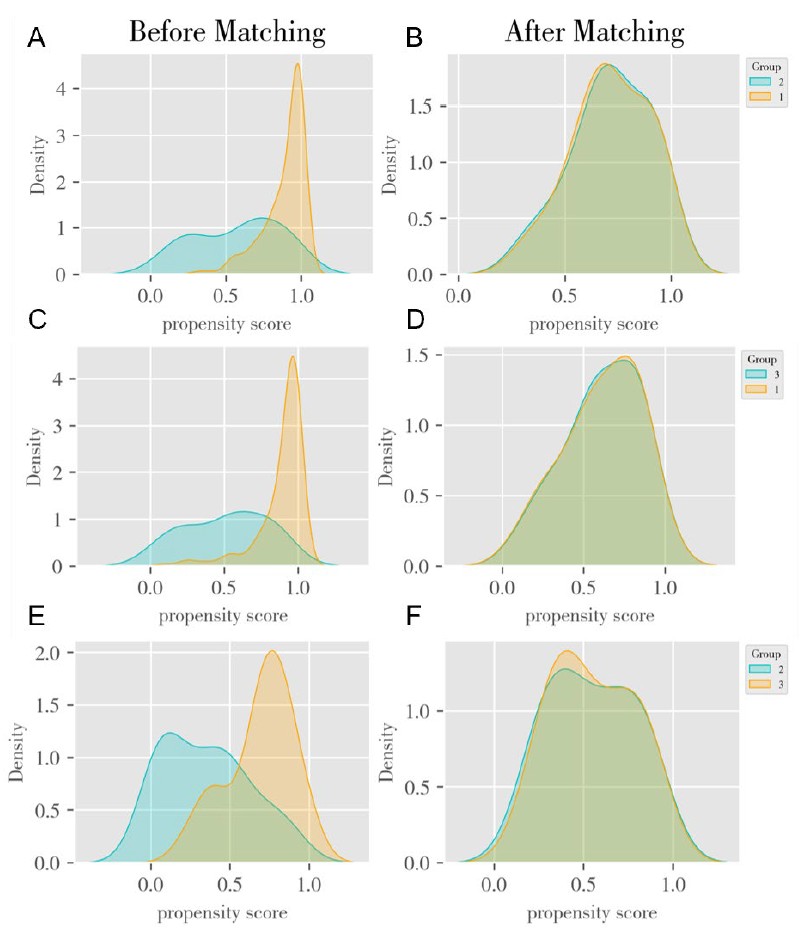

Supplement: Supplementary Figure 1 — The Love plots before and after PSM. (A, B): Love plots of the control group and the ICB + sRT group before and after PSM. (C, D): Love plots of the control group and the ICB + cRT group before and after PSM. (E, F): Love plots of the ICB + sRT group and the ICB + cRT group before and after PSM. Group 1: The control group. Group 2: The ICB + sRT group. Group 3: the ICB + cRT group. [file Image1.jpeg]
